# Supplementary material for: Autoantibodies to ACE2 and immune molecules are associated with COVID-19 disease severity
Source: Commun Med (Lond). 2024 Mar 15;4:47. doi: 10.1038/s43856-024-00477-z (PMC10943194; doi:10.1038/s43856-024-00477-z)
Supplement: Supplementary file 2 — Supplementary Information [file 43856_2024_477_MOESM2_ESM.pdf]

SUPPLEMENTARY INFORMATION

Autoantibodies to ACE2 and immune molecules are associated with COVID-19 disease severity

Eric S. Geanes, Rebecca McLennan, Cas LeMaster and Todd Bradley

<u>Table of contents</u>

Supplementary Table 1: Demographic information on human biospecimens used in the study.	Page 2
Supplementary Figure 1: ACE2 autoantibodies exist within healthy individuals.	Page 3
Supplementary Figure 2: Z-scores of ACE2 autoantibodies Ig subclass in severe COVID-19 individuals.	Page 4
Supplementary Figure 3: IgG, IgA, and IgM autoantibodies to cytokines, chemokines, and other immune factors in healthy and severe COVID-19 individuals.	Page 5

SUPPLEMENTARY TABLES

	**Healthy** (n=38)	**Mild COVID-19** (n=33)	**Severe COVID-19** (n=40)
**Age**	Median: 38 years old<br>Range: 22-75 years old	Median: 37 years old<br>Range: 25-67 years old	Median: 52.5 years old<br>Range: 24-77 years old
**Gender**	Male: 13<br>Female: 25	Male: 5<br>Female: 28	Male: 21<br>Female: 19
**Race**	White: 30<br>Asian: 3<br>Black/African American: 2<br>Hispanic/Latino; White: 2<br>Multiracial: 1	White: 28<br>Hispanic/Latino; White: 4<br>Asian: 1	White: 16<br>Black/African American: 6<br>Hispanic/Latino: 9<br>Hispanic/Latino; White: 8<br>Hispanic/Latino; White; Native American: 1

Supplementary Table 1: Demographic information on human biospecimens used in the study.

SUPPLEMENTARY FIGURES

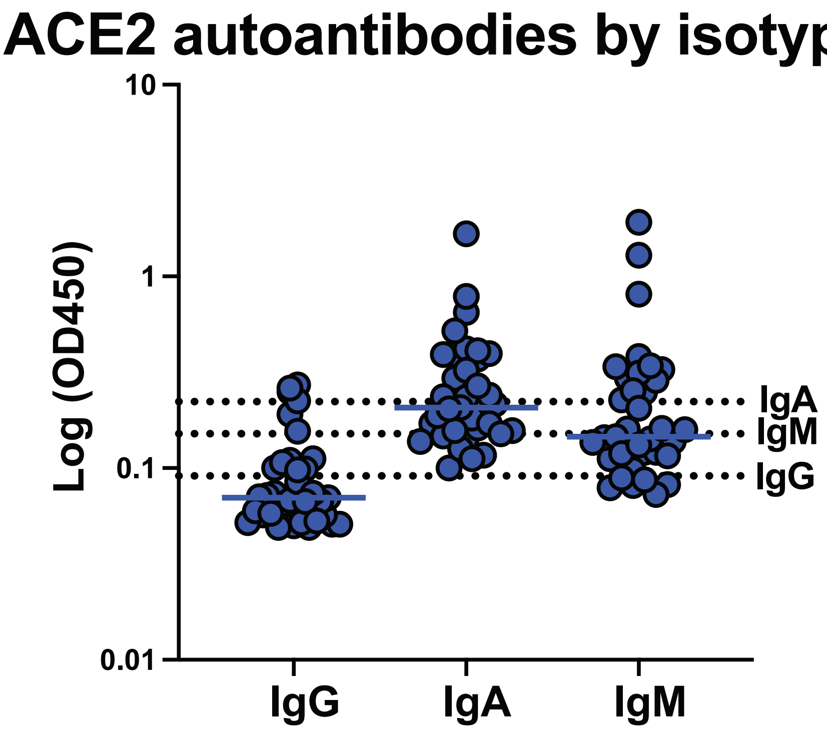

Supplementary Figure 1: **ACE2 autoantibodies exist within healthy individuals.** ACE2 autoantibodies for IgG, IgA, and IgM measured in plasma samples from healthy individuals with no prior SARS-CoV-2 infection by ELISA. Twice the measured background OD450 values were used to determine positivity cut-off for each independent Ig isotype (dashed lines). Each circle represents distinct individual, n=35. Lines represent the mean of all the samples for each isotype.

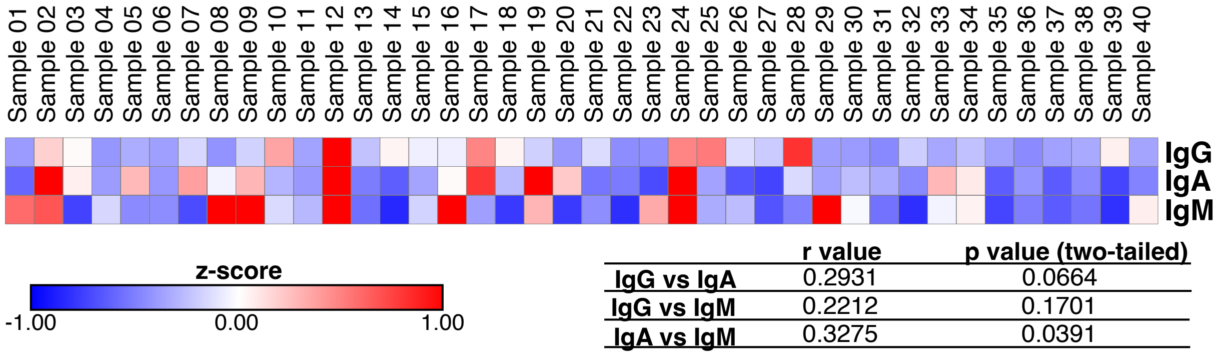

	r value	p value (two-tailed)
IgG vs IgA	0.2931	0.0664
IgG vs IgM	0.2212	0.1701
IgA vs IgM	0.3275	0.0391

Supplementary Figure 2: Z-scores of ACE2 autoantibodies Ig subclass in severe COVID-19 individuals. Heatmap of z-scores for each ACE2 autoantibody subtype (IgG, IgA, IgM). Table inset of Pearson correlations for each of the Ig comparisons.

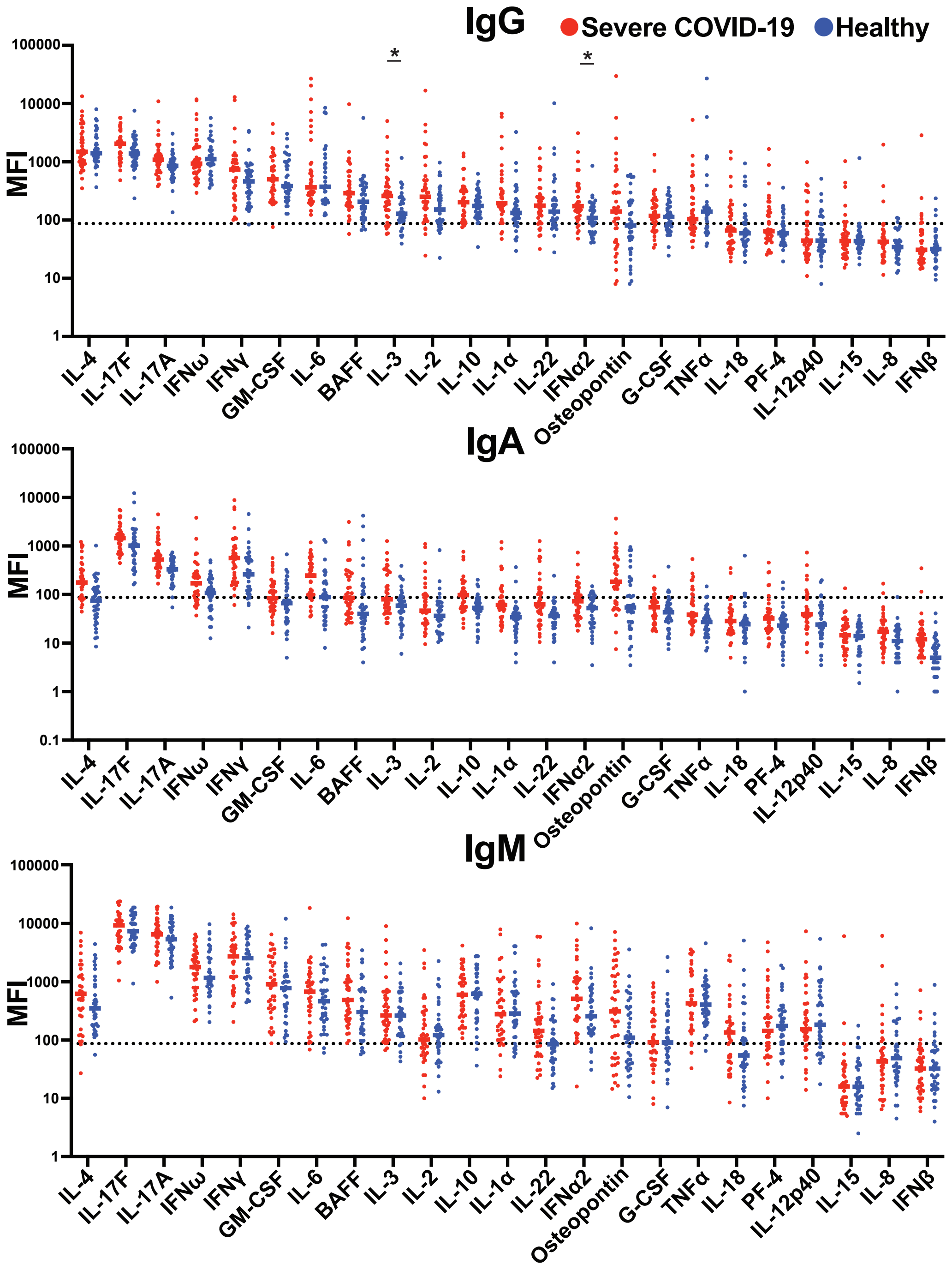

Supplementary Figure 3: **IgG, IgA, and IgM autoantibodies to cytokines, chemokines, and other immune factors in healthy and severe COVID-19 individuals**. Multiplex bead-based antibody binding assay measured the levels of autoantibodies against 23 cytokines, chemokines and immune molecules in severe COVID-19 (red, n=40) and healthy (blue, n=38) serum samples. Median Fluorescent Intensity (MFI) was calculated; background subtraction was used to remove nonspecific signal. The dashed line indicates a threshold determined by the sum of the mean and standard deviation for the negative control (i.e., beads without antigen). *, $p \leq 0.05$; Wilcoxon-Mann-Whitney.
