# Supplementary material for: Autoantibodies to ACE2 and immune molecules are associated with COVID-19 disease severity
Source: Commun Med (Lond). 2024 Mar 15;4:47. doi: 10.1038/s43856-024-00477-z (PMC10943194; doi:10.1038/s43856-024-00477-z)
Supplement: Supplementary file 3 — Description of Additional Supplementary Files [file 43856_2024_477_MOESM3_ESM.pdf]

Description of Additional Supplementary Files

File Name: Supplementary Data 1
Description: ACE2 peptide array raw data.

File Name: Supplementary Data 2
Description: Source Data
