# Supplementary material for: Autoantibodies to ACE2 and immune molecules are associated with COVID-19 disease severity
Source: Commun Med (Lond). 2024 Mar 15;4:47. doi: 10.1038/s43856-024-00477-z (PMC10943194; doi:10.1038/s43856-024-00477-z)
Supplement: Supplementary file 6 — Reporting Summary [file 43856_2024_477_MOESM6_ESM.pdf]

nature portfolio

Corresponding author(s): Todd Bradley

Last updated by author(s): 09/25/2023

Reporting Summary

Nature Portfolio wishes to improve the reproducibility of the work that we publish. This form provides structure for consistency and transparency in reporting. For further information on Nature Portfolio policies, see our Editorial Policies and the Editorial Policy Checklist.

Statistics

For all statistical analyses, confirm that the following items are present in the figure legend, table legend, main text, or Methods section.

n/a	Confirmed	
☐	☒	The exact sample size (*n*) for each experimental group/condition, given as a discrete number and unit of measurement
☐	☒	A statement on whether measurements were taken from distinct samples or whether the same sample was measured repeatedly
☐	☒	The statistical test(s) used AND whether they are one- or two-sided<br>*Only common tests should be described solely by name; describe more complex techniques in the Methods section.*
☒	☐	A description of all covariates tested
☐	☒	A description of any assumptions or corrections, such as tests of normality and adjustment for multiple comparisons
☒	☐	A full description of the statistical parameters including central tendency (e.g. means) or other basic estimates (e.g. regression coefficient) AND variation (e.g. standard deviation) or associated estimates of uncertainty (e.g. confidence intervals)
☒	☐	For null hypothesis testing, the test statistic (e.g. *F*, *t*, *r*) with confidence intervals, effect sizes, degrees of freedom and *P* value noted<br>*Give P values as exact values whenever suitable.*
☒	☐	For Bayesian analysis, information on the choice of priors and Markov chain Monte Carlo settings
☒	☐	For hierarchical and complex designs, identification of the appropriate level for tests and full reporting of outcomes
☒	☐	Estimates of effect sizes (e.g. Cohen's *d*, Pearson's *r*), indicating how they were calculated

Our web collection on statistics for biologists contains articles on many of the points above.

Software and code

Policy information about availability of computer code

Data collection	N/A
Data analysis	Graphs and statistics were analyzed using Graphpad prism verision 9.1. Heatmaps were generated using Morpheus, https://software.broadinstitute.org/morpheus.

For manuscripts utilizing custom algorithms or software that are central to the research but not yet described in published literature, software must be made available to editors and reviewers. We strongly encourage code deposition in a community repository (e.g. GitHub). See the Nature Portfolio guidelines for submitting code & software for further information.

Data

Policy information about availability of data

All manuscripts must include a data availability statement. This statement should provide the following information, where applicable:

- Accession codes, unique identifiers, or web links for publicly available datasets
- A description of any restrictions on data availability
- For clinical datasets or third party data, please ensure that the statement adheres to our policy

The data that support the findings of this study are available from the corresponding author, T.B., upon reasonable request.

Research involving human participants, their data, or biological material

Policy information about studies with human participants or human data. See also policy information about sex, gender (identity/presentation), and sexual orientation and race, ethnicity and racism.

Reporting on sex and gender	Sex and gender were based on self-reporting.
Reporting on race, ethnicity, or other socially relevant groupings	Race and ethnicity was based on self-reporting.
Population characteristics	Vaccine study participants include 33 mild SARS-CoV-2 seropositive and 38 healthy seronegative individuals. Seropositive population had a median age of 37 years old with a range of 25-67 years. There were 5 males and 28 females with race demographics of 28 white and 4 Hispanic/Latino; white, and 1 Asian. Seronegative population had a median age of 38 years old with an age range of 22-75 years. There were 13 males and 25 females with race demographics of 30 white, 3 Asian, 2 black/African American, 2 Hispanic/Latino; white, and 1 multiracial.<br>Severe SARS-CoV-2 participants include 40 individuals with a median age of 52.5 years old with a range of 24-77 years. There were 21 males and 19 females with race demographics of 16 white, 6 black/African American, 9 Hispanic/Latino, 8 Hispanic/Latino; white, and 1 Hispanic/Latino; Native American.
Recruitment	Pfizer-BioNTech Comirnaty® mRNA COVID-19 vaccine biospecimens were collected under a research study at Children's Mercy Kansas City. This study was reviewed and approved by the Children's Mercy IRB (#00001670 and #00001317). Participants self-enrolled after they had reviewed a study information letter and were given opportunity to ask questions. IRB waived written informed consent after these criteria were met.<br>Severe COVID-19 biospecimens were
Ethics oversight	Children's Mercy IRB (#00001670 and #00001317) and

Note that full information on the approval of the study protocol must also be provided in the manuscript.

Field-specific reporting

Please select the one below that is the best fit for your research. If you are not sure, read the appropriate sections before making your selection.

☒ Life sciences ☐ Behavioural & social sciences ☐ Ecological, evolutionary & environmental sciences

For a reference copy of the document with all sections, see nature.com/documents/nr-reporting-summary-flat.pdf

Life sciences study design

All studies must disclose on these points even when the disclosure is negative.

Sample size	No statistical method was used to predetermine the sample size via calculation. However, a minimum of 33 individual human samples were run for each condition in all experiments with the exception of 20 samples used for peptide microarray.
Data exclusions	No data was excluded in the analysis.
Replication	Assays were run with biological replicates of at least 20 or 33 individuals in singleton with standard controls.
Randomization	When selecting samples, seropositive and seronegative samples were randomized within each group. Severe samples were selected for 30-60 days post infection.
Blinding	Blinding was not applicable to the studies as identification of SARS-CoV-2 status was necessary for grouping.

Reporting for specific materials, systems and methods

We require information from authors about some types of materials, experimental systems and methods used in many studies. Here, indicate whether each material, system or method listed is relevant to your study. If you are not sure if a list item applies to your research, read the appropriate section before selecting a response.

Materials & experimental systems

n/a	Involved in the study
☐	☒ Antibodies
☒	☐ Eukaryotic cell lines
☒	☐ Palaeontology and archaeology
☒	☐ Animals and other organisms
☒	☐ Clinical data
☒	☐ Dual use research of concern
☒	☐ Plants

Methods

n/a	Involved in the study
☒	☐ ChIP-seq
☒	☐ Flow cytometry
☒	☐ MRI-based neuroimaging

Antibodies

Antibodies used	Jackson Immuno Research Goat anti-human IgG (Cat# 109-036-098 Lot# 149163), , Abcam Goat Anti-Human IgA alpha chain (Cat# ab97215, Lot# GR3373878-8), Abcam Goat Anti-Human IgM mu chain (Cat# ab97205, Lot# GR3396429-1)
Validation	Commercially purchased antibodies were validated by the manufacturers including assays or relevant publications (refer to catalog and lot numbers provided above).
